# Seroprevalence and factors associated with Hepatitis B virus infection among students in two senior high schools in the Krachi Nchumuru district in Ghana-A cross-sectional study

**DOI:** 10.1186/s13104-023-06624-4

**Published:** 2023-12-02

**Authors:** Patrick K. Nyambah, Richard Agjei, Bismark Sarfo

**Affiliations:** 1 Department of Epidemiology and Disease Control, School of Public Health, University of Ghana, P.O. Box 13, Legon-Accra, Ghana; 2 Health Directorate, P.O. Box 79, Kyinderi, Krachi Nchumuru District, Ghana; 3 Department of Health Administration and Education, University of Education, Winneba, Central Region Ghana

**Keywords:** Seroprevalence, HBV, Students, Circumcision, Ghana

## Abstract

**Background:**

There is paucity of hepatitis B virus (HBV) data among student populations although Ghana is HBV endemic. Screening and identification of factors associated with HBV transmission in schools will support the intervention in the elimination of the virus by 2030. This study assessed the seroprevalence and factors associated with HBV among students in two Senior High Schools in the Krachi Nchumuru District in Ghana.

**Methods:**

Through cross-sectional design and simple random sampling technique, 300 first-year students were enrolled from selected Senior High Schools. Structured questionnaires were used to collect data on demographic and exposure factors while rapid test kit was used to detect HBV infections. Chi-square/Fisher exact test and multivariable logistic regression were performed to determine the association between the variables at a 95% confidence interval and 0.05 significant level.

**Results:**

Seroprevalence of HBV was 14% (42/300) among the students. The prevalence was significantly (*p* = 0.001) higher in males 19.4% (34/175) than females 6.4% (8/125). 77.7% (233/300) were aware of HBV infection. Males who were circumcised were 4 times more likely to be infected with HBV (AOR = 4.09, 95%CI = 1.82–9.19) (*p* = 0.001) compared with those uncircumcised.

**Conclusion:**

HBV screening and education on hygienic genital circumcision practices must be prioritized in endemic countries.

**Supplementary Information:**

The online version contains supplementary material available at 10.1186/s13104-023-06624-4.

## Introduction

Hepatitis B virus infection is a global health concern. It is estimated that about 5% of the adult population in sub-Saharan Africa is infected with the hepatitis B virus [1].

Chronic hepatitis B infection is a major health issue in Ghana and attempts to estimate a nationwide prevalence of the disease has been challenging due to lack of primary data from some parts of the country. However, a systematic review of available published data indicates that the overall burden of hepatitis B virus (HBV) in Ghana is about 8.36% in the adult population, 14.30% in the adolescent population and 0.55% in children less than five years [2]. Hepatitis B infection attacks the liver and can cause acute and chronic diseases. In 2019 alone, about 820 people died from HBV infection of the liver causing cirrhosis and hepatocellular carcinoma [1]. The World Health Assembly adopted new strategies for the elimination of hepatitis B virus under the Sustainable Development Agenda 2030 [1]. Countries need to raise HBV awareness and scale up screening to prevent transmission of the virus. The virus can be transmitted through exposure to infected blood and body fluids, such as saliva, tattooing, piercing and needle stick injury [1, 3–6]. Transmission may also occur through the reuse of syringes or sharp objects such as razor blades [1, 6]. Additionally, sharing of toothbrushes, towels and cutlery can also lead to the transmission of the virus [5, 6]. It has been reported that risky behaviours common among students include unsafe daily practices like sharing cutlery, toiletries such as towels, and toothbrushes and re-use of razorblades [6]. Also some cultural and lifestyle practices like circumcision, scarification, tattooing, ear piercing and sexual activities which are very common among the youth significantly increase their risk of HBV infection [6–10].

Hepatitis B virus infection is vaccine-preventable and to prevent HBV transmission in childhood, the HBV vaccine was introduced in the expanded program on immunization (EPI) in many sub-Saharan African countries [3]. It was introduced in Ghana in 2002 [11]. Hepatitis B Virus infection however remains high in the country with varying prevalence despite the introduction of the vaccine [12].

The prevalence of HBV infection is much higher in rural communities than in urban areas, and the Krachi Nchumuru has a rural population of 69.9% according to the 2021 population and housing census [13].

Although anecdotal evidence has suggested high prevalence of HBV in the district, there is no comprehensive study to assess the actual prevalence of the disease in the area. In a previous pilot study that we conducted among the second- and third-year students of Nchumuru Community High School and St. Theresa’s Vocational Institute, we observed a prevalence of about 15.5% (Nyambah and Sarfo, unpublished), although we did not assess the factors associated with the virus transmission. With increasing number of students in Senior High Schools as a result of the recently implemented free Senior High School policy in Ghana, the number of students in schools and hostels are bound to increase. Meanwhile HBV vaccination and screening of students are not requirements for students’ enrolment into any of the Senior High schools in Ghana. Hence the risk of HBV transmission among these students could be high.

Screening of HBV in the various institutions and identifying the factors associated with transmission of the virus will support the overall targeted intervention strategy in the elimination of HBV by 2030. Therefore, this study assessed the seroprevalence and factors such as socio-demographic characteristics, sharing of towels, toothbrushes, cutlery, razor blades, ear piercing and genital circumcision that could influence Hepatitis B Virus transmission among students in two Senior High Schools, namely the Nchumuru Community Senior High School and the St. Theresa Vocational Institute in the Krachi Nchumuru District (KND) in the Oti region of Ghana.

## Methods

### Study area

The study was conducted in two selected Senior High Schools (SHS) in Chinderi in the Krachi Nchumuru District (KND) in the Oti Region of Ghana. The District is predominantly rural [13] and there are only four SHS in the district. The two selected schools are all located in the District capital (Kyinderi) and attract adolescents and young adults from neighbouring towns and villages who want to further their education after completion of their Junior High School programs. The schools were purposely selected to represent SHS in the district and also against the background that population movement and congregation is associated with HBV transmission and spread.

### Study design

**Fig. 1:**
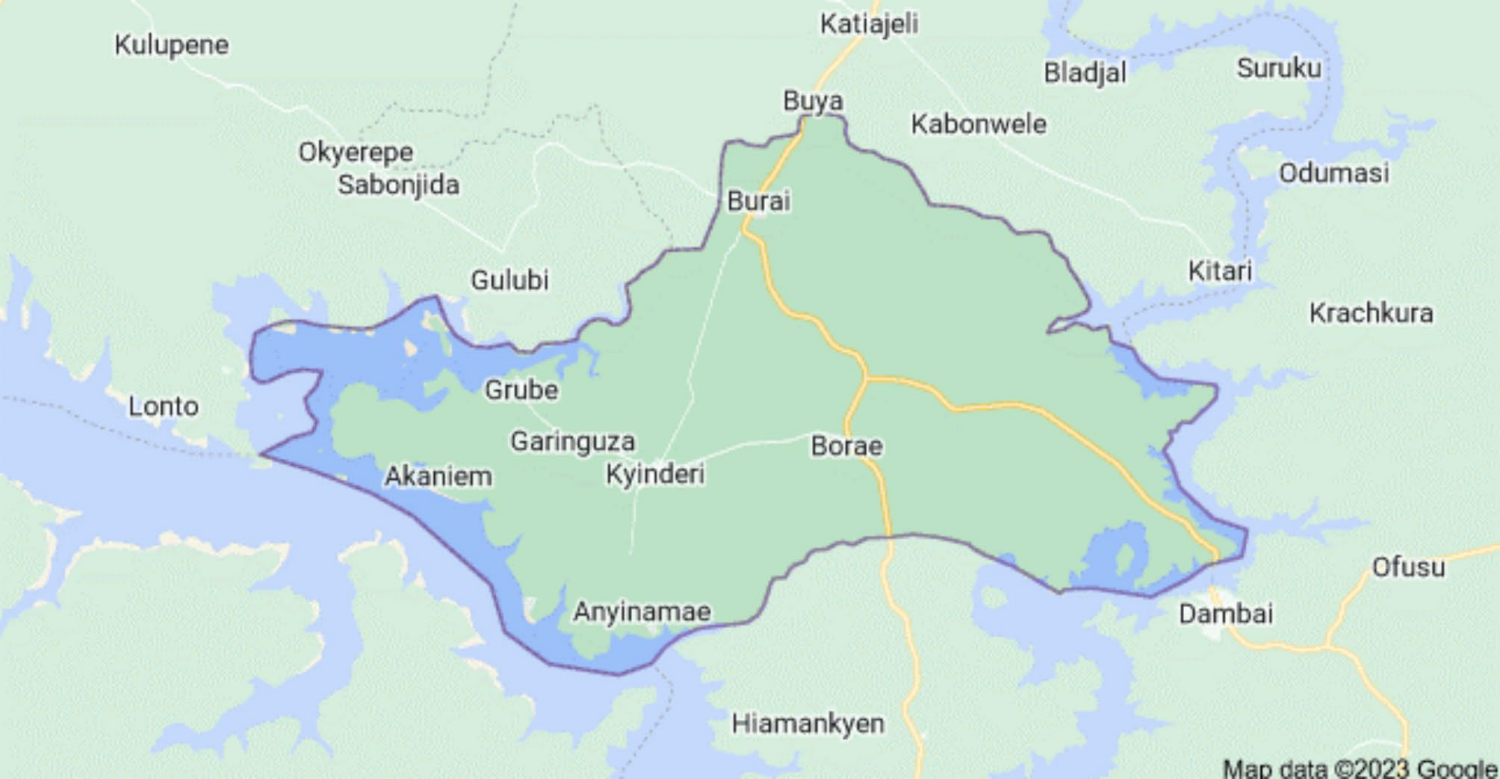
Map of Krachi Nchumuru District (KND) in the Oti Region of Ghana showing the District capital, Kyinderi (Chinderi) and adjoining towns (Source: Google map [14])

This is a cross-sectional study conducted among students enrolled in two Senior High Schools at Kyinder (Chinderi) (Fig. 1) in the Krachi Nchumuru District.

### Study population

The study population was made up of first year Senior High School students enrolled from the Nchumuruman Community Senior High School and the St. Theresa’s Vocational Institute, all at Chinderi (Fig. 1) in the KND in the Oti region of Ghana.

### Inclusion criteria

All first-year students both males and females at the participating schools were eligible to be included in the study.

### Exclusion criteria

Students in the second- and third year and those who did not assent or whose parents did not consent were excluded from the study.

### Nchumuruman community senior high school

The Nchumuruman Community Senior High School was established at Chinderi (Fig. 1) in 2013 and in 2016 it was upgraded as one of the Government of Ghana and World Bank sponsored community Day schools in Ghana. The school admits both girls and boys from the various towns within the KND as shown in Fig. 1, and outside of the district, and the student population of those in the first year at the time of the study was 294.

### St. Theresa’s vocational institute

The St. Theresa’s Vocational Training Institute is a Mission School established by the Catholic Church in 1984 in KND. The main mission of the Institute is to provide employable skills to Junior High School and Senior High School leavers through competency-based apprenticeship, master craftsmanship and general career development. In 2001, the National Vocational Training Institute (NVTI) of Ghana absorbed the St. Theresa’s Vocational Institute as a Public Institution and it is currently being run as a Boarding and Day School with a total first year student enrolment of 76 at the time of the study.

### Sample size estimation

A study conducted in the Central African Republic in a cohort of students reported an overall prevalence of 42.3% for antibody to hepatitis B core antigen, and 15.5% for hepatitis B surface antigen (HBsAg) [15]. Using HBsAg prevalence of 15.5% in this study and the Cochrane formula [16] for estimating sample size in cross-sectional design with percentage margin of error taken as 5%= 0.05, Z score of 1.96, and a 10% non-response rate, yielded an estimated sample size of 228. An additional 72 students who expressed interest in knowing their hepatitis B status assented to participate in the study after their parents have consented, making the overall sample size of the study to be 300 participants.

### Sampling technique

Proportionate to size calculation was used to determine the number of participants to be enrolled from each school. There are more first year students in Nchumuruman SHS (294) than St. Theresa’s Vocational Institute (76). A simple random sampling technique was used to select the participants from each school.

### Enrollment of students’ participants

To give each student an equal chance of participating in the study, the school register which is a compilation of students’ names with numbers in each school was used as a sample frame. Eligible students were selected using Google based random number generator software program. Random generator program can generate one or many random numbers within a defined framework. For Nchumuruman SHS, the framework was set between 1 and 294 while that for the St. Theresa’s Vocational Institute was between 1 and 76. Any student whose number in the register corresponded with a randomly generated number was selected and enrolled into the study. This procedure was repeated until all the 300 students were enrolled into the study.

### Data collection techniques and tools

A structured questionnaire (Additional file 1: Appendix A) which has been categorized into sections such as socio-demographic factors, prevalence of Hepatitis B virus infection, risk factors for Hepatitis B virus infection, and knowledge of Hepatitis B virus infection, was used to interview the students to collect data on those factors. For instance, the students were asked to answer questions on risk factors such as sharing of towels, toothbrushes, cutlery, razor blades, ear piercing, genital circumcision etc. which are very common among them. Subsequently, the HBV surface antigen test was used to determine the seropravelence of HBV among these students.

### Testing the participants for HBV infection

The One Step HBsAg whole blood/serum/plasma test for qualitative assessment of hepatitis B surface antigen (HBsAg) in human, manufactured by Wondof Biotech Company Ltd in Guangzhou China was used to determine HBV infection among the students from the two schools (Nchumuruman SHS and St. Theresa’s Vocational Institute).

During the testing, thumb of each participant was wiped with an alcohol pad and a strip of blood was taken by pricking the thumb with a sterilized lancet needle. Using a dropper provided in the kit by the manufacturer, about 2–3 drops of blood was transferred onto the cassette which contains the hepatitis B virus surface antigen. Following the manufacturer’s instructions, the results were read within 15 min. The HBsAg test kit has a sensitivity of 96.2% and specificity of 99.3%. Standard operating procedures through aseptic means were followed to avoid pathogen contamination during finger pricking and blood sample collection from the participants for the hepatitis B test. Personal protective equipment including gloves was worn throughout the procedure and a new sterilized lancet needle was used for each participant. As far as possible, respiratory, and environmental hygiene were consistently observed during the testing and the waste material that were generated were properly disposed.

Prior to testing the students, an arrangement was made with the St. Luke Clinic at Chinderi for management of those who will test positive for HBV. Following this arrangement, those who tested positive for HBV were referred to the clinic for further management. In general, all the students were educated and counselled on Hepatitis B transmission and were also directed to go for the Hepatitis B vaccine at the clinic.

### Data management and analysis

Data collected was checked for completeness and clarity. Data were entered into Excel before importing into STATA 15 software for coding, cleaning and analysis. Descriptive analysis for seroprevalence of Hepatitis B Virus infection and socio-demographic characteristics including all the exposure factors were performed. Person’s chi-square and Fisher exact test, as well as bivariate analysis were performed to explore the association between the socio-demographic and exposure factors such as sharing of cutlery, towels, brushes, razor blades, genital circumcision, ear piercing etc. and hepatitis B virus infection. Multivariable logistic regression was performed and adjusted for variables that were significant at the bivariate analysis stage. Statistical significance was set at 0.05 at 95% confidence intervals.

### Ethical consideration

The study received ethical approval from the Ghana Health Service Ethics Committee with approval number GHS-ERC: 048/12/17. Permissions were obtained from the two selected Senior High Schools authorities before the commencement of the study. Also, before signing the consent and assent forms, each parent and participant was educated on the purpose, the risk, the testing procedure, and the benefits of the research. Parents consented on their wards behalf and wards also assented on their own to participate in the study The participants were informed that they could withdraw from the study at any stage without any consequences. Questionnaires were administered to students after they consented, and finger-prick blood samples were taken from them for HBV testing. Pretesting of the questionnaires were done at a different Senior High School (Oti Senior High School) in the Krachi East District to assess the consistency and clarity of the questionnaire. Privacy was ensured during the testing period since it was done in an enclosed setting which allowed only one student at a time.

### Data quality assurance

All data collectors were properly and adequately trained in how to collect data for the study including how to communicate effectively with the participants. Data that were collected were assessed to make sure that adequate information on the questionnaire have been collected. Double entries were done, to minimize data entry errors and also to address any inconsistencies.

### Data protection plan

Privacy and confidentiality of the participants’ data were ensured at all stages of the study. Administration of the study questionnaire and testing for Hepatitis B virus infection were done in an enclosed room with each participant at a time. Unique identification numbers were assigned to the participants instead of their names and the questionnaires were kept in a safe and locked, with the key available to only key members of the research team. Electronic copy of the study data was stored on a password protected computer.

## Results

Table 1 shows that, majority of the respondents were within the age category 15–20 years, representing 90.3%, (271/300). Only very few (0.7%) were more than 25 years. The respondents were dominated by males representing 175/300 (58.3%) as compared to their female counterparts which recorded 125/300 (41.7%). 293/300(97.7%) of the respondents were single, whiles only 1.0% (3/300) were married.

**Table 1:** Demographic characteristics of the study participants

Variables	Frequency(n = 300)		Percentage (%)
**Age**			
15–20	271		90.3
21–25	27		9.0
> 25	2		0.7
**Sex**			
Male	175		58.3
Female	125		41.7
**Marital Status**			
Single/Never Married	293		97.7
Married	3		1.0
Separated/Divorced	4		1.3
**Residential Status**			
On Campus	59		19.7
Off Campus	241		80.3
**Religious Affiliation**			
Christians	270		90.0
Moslems	24		8.0
Traditionalist	6		2.0
**Institution**			
Nchumuruman SHS	238		79.3
St. Theresa’s Vocational Institute	62		20.7

**Fig. 2:**
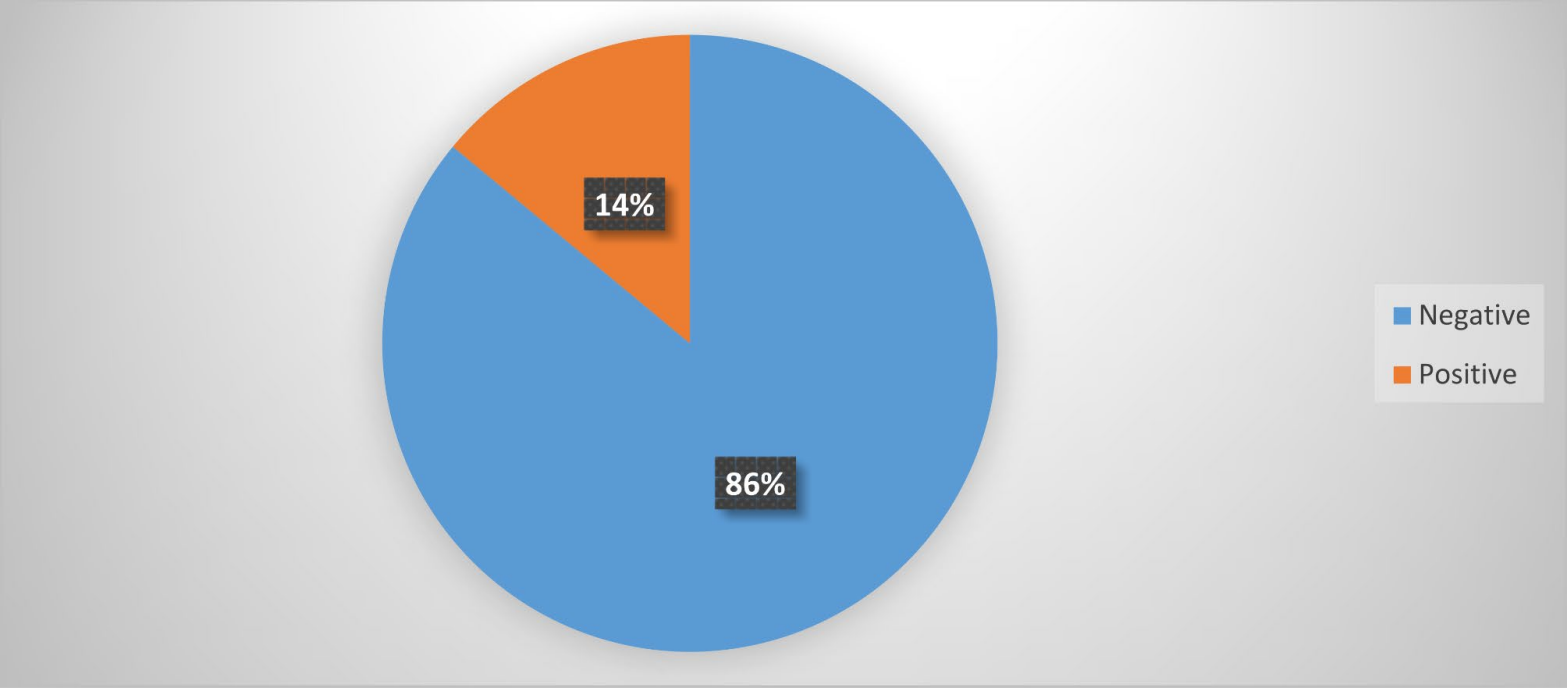
Prevalence of Hepatitis B Virus infection among respondents

As demonstrated in Fig. 2, eligible first-year students from Nchumuruman Senior High School as well as St. Theresa’s Vocational institute were screened for Hepatitis B Virus infection out of which 14% (42/300) tested positive and 86% (258/300) were negative for the test.

**Fig. 3:**
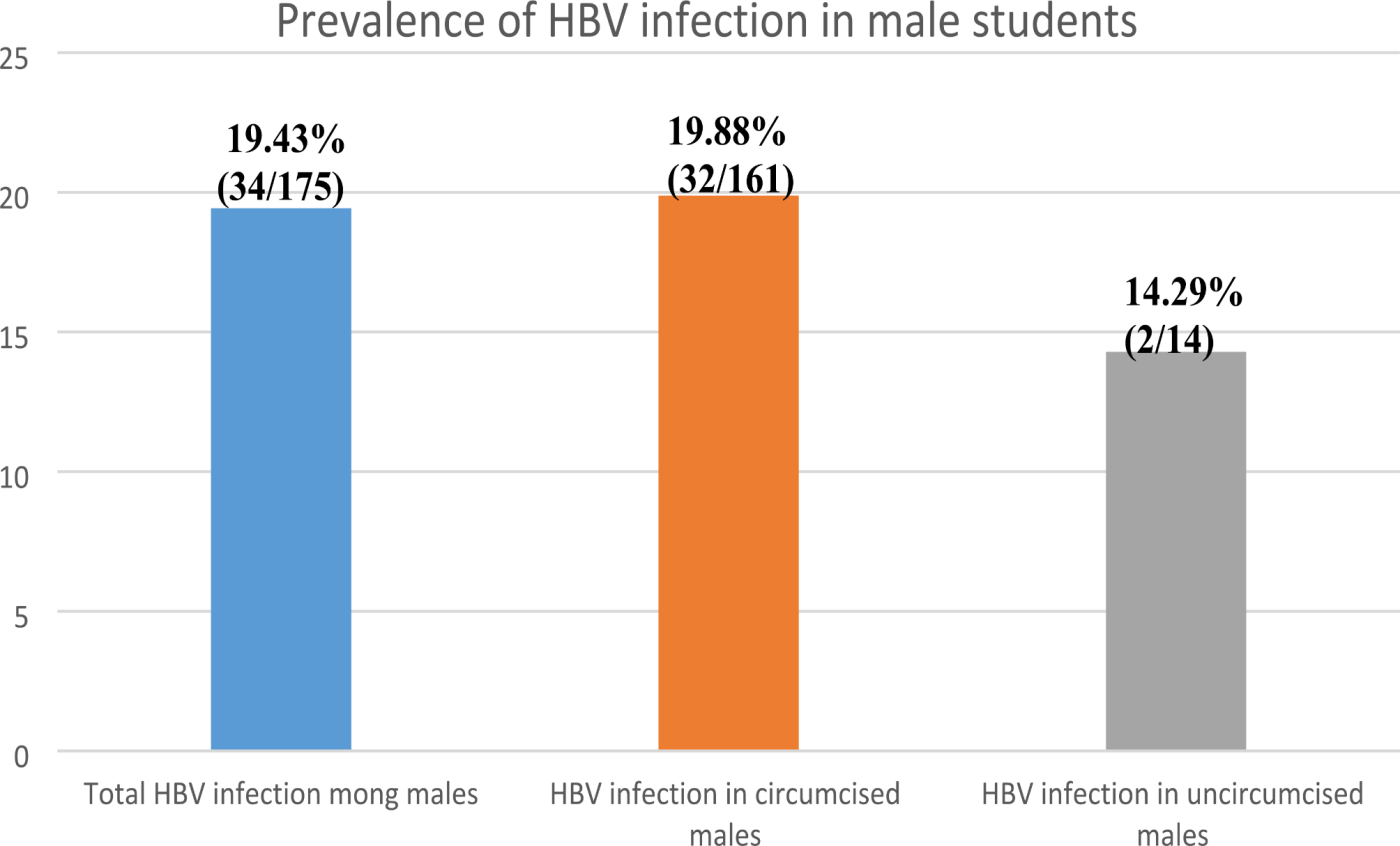
Prevalence of hepatitis B virus infection among male participants

Figure 3 shows that there were 175 males in the study with 161 who have circumcised and 14 had not uncircumcised. Overall, 19.43% (34/175) of the male participants tested positive for HBV, 19.88% (32/161) tested positive among circumcised males while 14.29% (2/14) tested positive for HBV among uncircumcised males.

As indicated in Table 2, the prevalence of Hepatitis B Virus infection among Senior High School students varies in age groups. Students within the age group 15–20 years have the highest number (33) of HBV infection compared with other age categories. There was statistical significance (p = 0.032) in the association between age and HBV positive test. The prevalence of Hepatitis B Virus infection was high in males 34/175 (19.4%) as compared to 8/125 (6.4%) females and this was statistically significant (p < 0.05).

**Table 2:** Socio-demographic factors and the prevalence of Hepatitis B Virus infection among SHS students

Variables	HBV- No.(%)	HBV + No.(%)	Pearson Chi2 /Fisher exact	P-value
**Age** ^**^			**8.7904**	^*^ **0.032**
15–20	238(87.8%)	33(12.2%)		
21–25	19(70.4%)	8(29.6%)		
> 25	1(50%)	1(50%)		
**Sex**			**10.28**	^*^ **0.001**
Male	141(80.6%)	34(19.4%)		
Female	117(93.6%)	8(6.4%)		
**Marital Status**			1.1668	^**a**^0.558
Single/Never Married	251(85.7%)	42(14.3%)		
Married	3(1.2%)	0(0%)		
Separated/Divorced	4(1.6%)	0(0%)		
**Residential Status**			0.2782	0.598
On Campus	52(88.1%)	7(11.9%)		
Off Campus	206(85.5%)	35(14.5%)		
**Religious Affiliation**			3.8344	^**a**^0.147
Christians	231(85.6%)	39(14.4%)		
Moslems	23(95.8%)	1(4.2%)		
Traditionalist	4(66.7%)	2(33.3%)		
**Institution**			0.172	1.8612
Nchumuruman SHS	208(87.4%)	30(12.6%)		
St. Theresa’s Vocational Institute	50(80.6%)	12(19.4%)		

^a^=Fisher exact, ^*^Statistical significance, *p* < 0.05

Table 3 shows that, there was a statistically significant relationship between the prevalence of Hepatitis B Virus infection among SHS students and male circumcision (*p* < 0.05) as well as piercing of ears (*p* < 0.05). The rest of the observed risk factors by variable measure among SHS students did not show statistical significant relationship with Hepatitis B Virus infection.

**Table 3:** Prevalence of Hepatitis B Virus infection and it associated risk factors among students

Variable	HBV- No.(%)	HBV + No.(%)	Pearson Chi2/Fisher	P-value
**Sharing Toothbrush**			0.463	0.793
Yes	78(87.6%)	11(12.4%)		
No	180(85.3%)	31(14.7%)		
**Sharing Sponge and Towel**			1.5127	0.219
Yes	155(88.1%)	21(11.9%)		
No	103(83.1%)	21(16.9)		
**Sharing Chewing sticks**			0.7979	0.372
Yes	52(89.7%)	6(10.3%)		
No	206(85.1%)	36(14.9%)		
**Sharing spoon**			0.7411	0.389
Yes	193(85%)	34(15%)		
No	65(89%)	8(11%)		
**Sharing blades**			0.2137	0.644
Yes	137(85.1%)	24(14.9%)		
No	120(87)	18(13%)		
**Sharing Shaving Sticks**			1.003	0.317
Yes	61(89.7%)	7(10.3%)		
No	197(91.8%)	35(15.1%)		
**Sharing Knife**			2.6778	0.102
Yes	191(84.1%)	36(15.9%)		
No	67(91.8%)	6(8.2%)		
**Sharing Needles**			0.7135	0.398
Yes	111(84.1%)	21(15.9%)		
No	147(87.5%)	21(12.5%)		
**Sexual relationship**			0.00017	0.967
Yes	165(85.9%)	27(14.1%)		
No	93(86.1%)	15(13.9%)		
**Sexual partners**			0.0086	0.996
One	127(85.8%)	21(14.2%)		
Two	19(86.4%)	^a^3(13.6%)		
More than two	19(86.4%)	^a^3(13.6%)		
**Using condom**			2.786	0.097
Yes	64(91.4%)	6(8.6%)		
No	101(82.8%)	21(17.2%)		
**Tribal Marks**			0.5175	0.472
Yes	126(87.5%)	18(12.5%)		
No	132(84.6%)	24(15.4%)		
**Tattoos**			0.66	^a^0.417
Yes	^a^4(100%)	^a^0(0%)		
No	141(81.5%)	32(18.5%)		
**Ear piercing**			**6.8644**	^*^ **0.009**
Yes	117(92.1%)	10(7.9%)		
No	142(81.5%)	32(18.5%)		
**Male circumcision**			**9.9641**	^*^ **0.002**
Yes	129(80.1%)	32(19.9%)		
No	129(92.8%)	10(7.2%)		
**Female circumcision**			0.66	^a^0.417
Yes	^a^4(100%)	^a^0(0%)		
No	254(85.8%)	42(14.2%)		
**Blood transfusion**			1.327	0.1716
Yes	16(88.9%)	^**^2(11.1%)		
No	242(85.8%)	40(14.2%)		

^a^=Fisher exact, ^*^Statistical significance, *p* < 0.05

The results in Table 4 show that male circumcision was the only variable which was significantly associated with HBV infection among the SHS students. In the crude model, students who underwent male circumcision were 3.2 times more likely to be infected with HBV (COR = 3.2, 95%CI = 1.51–6.78) (*p* = 0.002) as compared to those who were not circumcised. After adjusting for other observed covariates, male circumcision was still significantly associated with HBV infection among the students where male students who were circumcised are about 4 times more likely to be infected with HBV (AOR = 4.09, 95%CI = 1.82–9.19) (*p* = 0.001) compared with those who were not circumcised.

**Table 4:** Hepatitis B Virus infection among SHS students and it associated factors using bivariate and multiple regression analysis

Variable	N	Unadjusted OR	P-Value 95% CI	Adjusted OR	P-Value 95% CI
**Age**					
21–25	27	1		1	
15–20	271	1.32	0.50 (0.59–2.98)	1.13	0.79 (0.47–2.72)
> 25	2	8.78	0.14 (0.50-152.71)	6.74	0.23 (0.30-152.26)
**Sex**					
Female	125	1			
Male	175	1.67	0.57 (0.89–3.45)	1.43	0.21 (0.68–3.45)
**Male Circumcision**					
No	139	1		1	
Yes	161	**3.2**	^*^ **0.002** **(1.51–6.78)**	**4.09**	^*^ **0.001** **(1.82–9.19)**
**Sharing spoon**					
No	73	1		1	
Yes	227	1.43	0.39 (0.63–3.64)	1.71	0.25 (0.69–4.24)
**Ear piercing**					
**No**	**173**	**1**		**1**	
**Yes**	**127**	**1.67**	**0.09** **(0.34–4.21)**	**1.78**	**0.15** **(0.45–5.03)**
**Sharing blades**					
No	138	1		1	
Yes	161	1.17	0.64 (0.60–2.26)	1.13	0.75 (0.54–2.33)
**Sexual relationship**					
No	108	1		1	
Yes	192	1.01	0.97 (0.51–2.70)	1.14	0.74 (0.52–2.54)
**Sharing needle**					
No	168	1		1	
Yes	132	1.32	0.40 (0.69–2.54)	1.13	0.75 (0.54–2.34)
**Sharing knife**					
No	73	1		1	
Yes	227	2.10	0.11 (0.85–5.22)	2.62	0.07 (0.93–7.40)

^*^Statistical significance, *p* < 0.05

## Discussion

This study has demonstrated that the seroprevalence of Hepatitis B Virus infection among the Senior High School students in the Krachi Nchumuru District was high. Students between the ages of 15–20 years recorded the highest prevalence, and the HBV infection was higher in males than females. Majority of the male students who have HBV infection have circumcised, and male circumcision was the only factor which was statistically and significantly associated with HBV infection among the students. Although several behavioral known risk factors such as ear piercing, sharing of blades, needles and knifes and other sexual practices, associated with HBV were assessed in this study, they were not statistically significantly associated with HBV infection.

In terms of endemicity, the prevalence of HBV infection in this study is consistent with a systematic review report which estimated that the prevalence of HBV among adolescent population in Ghana is 14.3% [2]. This finding is also in line with a study conducted in Ghana which recorded a prevalence of 10.8% of HBV infection among Voluntary Blood Donors (VBDs), 12.7% for replacement blood donors (RBVs), 13.1% among pregnant women and 6.7% among medical laboratory science students (MLSSs) in the University of Health and Allied Sciences in Ghana [15, 16]. Meanwhile in Togo, Ekouevi and colleagues (2017) [6] recorded a prevalence of 4.6% HBV infection among students from the University of Lomé, while a similar research conducted in Nairobi Kenya, recorded a prevalence of 3.0% HBV infection among the youth in public high schools [17]. These are far below the reported HBV prevalence in this study, and differences in demographic characteristics and other factors including traditional and cultural practices such as scarification for tribal marks could have contributed to the high burden and the disparities in HBV prevalence.

With regards to the HBV prevalence among the age categories, the reported high prevalence among the 15–20 years somewhat contradicts the age burden of HBV infection in the country as reported by Abesig and colleagues [2] in their 2020 systematic review. The sampled populations coupled with the geographical location of the study area could have contributed to this apparent difference in the age burden of HBV infection.

The high HBV burden among this age category and the students in general in this rural part of Ghana underscores the need for scaling up testing and intensifying HBV vaccination in the area. The study with other previous reports confirm the lack of any HBV program in the area. Therefore, Government must come up with a policy to either subsidize HBV vaccine or make it free for those living in rural and hard to reach communities with high HBV burden. Currently, HBV vaccination must be paid out of pocket in Ghana, therefore people with low incomes living in the rural communities may not prioritize HBV vaccination over other competing economic expenditures.

This study also showed that there was statistically significance between sex and HBV infection with males recording higher prevalence than females. This is consistent with a study conducted in Tamale in the Northern Region of Ghana which indicated that sex as a variable was a determinant factor for HBV infection among blood donors [18]. Furthermore, a study in Kintampo in the Bono East Region of Ghana among blood donors recorded a prevalence of 9.7% HBV infection among males as compared to 8.5% prevalence in their female counterparts [19]. Males recording high HBV infection than females may be attributable to many factors including the fact that males are known to indulge in risky behaviors than females, which can predispose them to HBV infection. Meanwhile, apart from only 2 respondents, all the male study participants have circumcised.

In the current study, in addition to male students recording high prevalence of HBV infection, male circumcision was also found to be significantly associated with the HBV infection. When other observed covariates were controlled for in this study, male students who were circumcised were 4 times more likely to be infected with hepatitis B viral infection.

Male circumcision has been reported to prevent the transmission of sexually transmitted infections [20–22], although the American Academy of Pediatrics Task Force on Circumcision in their 1999 [23] report shows that such evidence is rather conflicting. Notwithstanding this, studies have shown that male circumcision provides partial protection in men against HIV and some sexually transmitted infections (STIs) [24–28]. But the mode of the circumcision procedure if not properly performed can also lead to some blood borne infections. A study conducted in Turkey has shown that circumcision performed by non-medical personnel such as traditional practitioners and barbers has been associated with the prevalence of viral hepatitis [28] while in Uganda, in a Muslim community, it was common for groups of 3–10 children to be circumcised using a single instrument [29], which can expose them to infections.

Indeed, the finding from this study corroborates the result of a study by Eke and colleagues (2015) [30] which reported high HBV infection among adolescent circumcised males in Enugu in Nigeria.

Krachi Nchumuru district is predominantly rural with very few health centers, clinics and Community Health-Based Planning Services (CHPS) compounds. Currently the district has no hospital, so health emergencies are referred to the Kete-Krachi Hospital which is about 65 km from the Krachi Nchumuru district. Against this background, traditional and home-based circumcision of males is very common in the area, although some circumcisions do occur at the health facilities. Since male circumcision can also be associated with religious and cultural beliefs, some would avoid going to the facilities to be circumcised and would prefer the traditional circumcision. Home-based or traditional circumcision can be unhygienic when razor blades or instruments that are used to circumcise are not sterilized. This can lead to infections and transmission of blood borne diseases including HIV and hepatitis. The findings from this study could be attributed to unhygienic circumcision which is very common with home-based and traditional circumcision practices, although some health facilities may also be culpable of these practices. It is plausible that the males who tested positive for HBV could have gotten the infection at childhood or at younger ages when they got circumcised. Although the data for this study was not disaggregated into health facility and home-based circumcision, it did provide a strong association between genital circumcision and HBV infection which warrants urgent public health attention.

The findings from this study should be interpreted within the context of the limitations that information on routine immunization against Hepatitis B was not collected from the students. Further, information on traditional, home and facility-based circumcision were not collected because the students could not recall when and where they were circumcised.

Notwithstanding the above limitations, the findings from this study provide very critical information which would be useful for intervention policies in the elimination of hepatitis B virus infection under the Sustainable Development Agenda 2030.

## Conclusions

This study has demonstrated high seroprevalence of hepatitis B virus infection among first year Senior High School students which is significantly associated with male circumcision. Therefore, there is urgent need for identification and registration of male genital circumcision practitioners in communities where HBV infection is high. This will provide the opportunity for health authorities to organize workshops and training about hygienic way of circumcision to prevent HBV transmission. >Also, school authorities must ensure that newly qualified SHS students are screened for HBV and fully immunized before admission. The report of this study was shared with School Authorities and this recommendation is currently being practiced. Additionally, Ministries of Health should incorporate HBV screening, immunization, and management into National Health Insurance Schemes to reduce the disease burden among young adults such as Senior High School students. All these would be in line with promoting partnerships, mobilizing resources, and formulating evidence-based policy and data action for the elimination of hepatitis B virus infection by 2030.

### Electronic supplementary material

Below is the link to the electronic supplementary material.

**Additional file 1: Appendix A**: Questionnaire

**Additional file 2: Supplementary Data 1**: Logistic Regression assumptions

## Data Availability

The datasets used and/or analysed during the current study are available from the corresponding author on reasonable request.

## List of abbreviations

AOR: Adjusted odds ratio
COR: Crude odds ration
EPI: Expanded Program on Immunization
HBsAg: Antibodies to Hepatitis B Surface Antigen
HBV: Hepatitis B Virus
NHIS: National Health Insurance Scheme
NVTI: National Vocational Training Institute
SHS: Senior High School
WHO: World Health Organization
