# Supplementary material for: Seroprevalence and factors associated with Hepatitis B virus infection among students in two senior high schools in the Krachi Nchumuru district in Ghana-A cross-sectional study
Source: BMC Res Notes. 2023 Dec 2;16:358. doi: 10.1186/s13104-023-06624-4 (PMC10693693; doi:10.1186/s13104-023-06624-4)
Supplement: Supplementary file 1 — Additional file 1: Appendix A: Questionnaire [file 13104_2023_6624_MOESM1_ESM.docx]

## Appendix A: QUETIONNAIRE

SCHOOL OF PUBLIC HEALTH

COLLEGE OF HEALTH SCIENCES

UNIVERSITY OF GHANA, LEGON

**PROJECT TITLE;** FACTORS ASSOCIATED WITH HEPATITIS-B VIRUS INFECTION AMONG SENIOR HIGH SCHOOL STUDENTS IN THE KRACHI NCHUMURU DISTRICT OF THE VOLTA REGION.

**Section-1 Socio-Demographic Factors.**

1. Age:

(a) 15-20 [ ]

(b) 21-25 [ ]

(c) >25 [ ]

1. Sex:

(a) Male [ ]

(b) Female [ ]

1. Marital Status

(a) Single/Never Married [ ]

(b) Married [ ]

(c) Separated/ Divorced [ ]

1. Place of Residence

(a) Urban [ ]

(b) Rural [ ]

1. What is your religious affiliation?

(a) Christian [ ]

(b) Moslem [ ]

(c) Traditionalist [ ]

(d) Atheist [ ]

(e) Buddhist [ ]

1. Name of Institution

(a) Nchumuruman SHS [ ]

(b) St. Theresa’s Vocational School [ ]

7. What is your residential status in your school.

(a) On campus/Boarder [ ]

(b) Off campus/ Day student [ ]

**Section-2 Prevalence of Hepatitis B Virus Infection**

1. Hepatitis B Virus Test Result

(a) Negative [ ]

(b) Positive [ ]

**Section 3**-**Risk Factors**

1. Tick the following items you have ever shared with your colleagues before?

(a) Toothbrush [ ]

(b) Sponge and Towel [ ]

(c) Chewing Sticks [ ]

(d) Spoon [ ]

(e) None of the above [ ]

1. Which of the following sharps have you ever shared with your colleagues?

(a) Blades [ ]

(b) Shaving Sticks [ ]

(c) Knife [ ]

(d) Needles [ ]

1. Have you ever been or are you in a sexual relationship?

(a) Yes [ ]

(b) No [ ]

1. If Yes, how many sexual partners do you have?

(a) One [ ]

(b) Two [ ]

(c) More than two [ ]

1. Do you usually use Condom during sexual intercourse?

(a) Yes [ ]

(b) No [ ]

1. Which of the following do you have? **Please observe for tribal marks/tattoos and ear piercing and tick (a) presence (b) absence**
2. Tribal Marks [ ]
3. Tattoos [ ]
4. Ear Piercing [ ]
5. None of the above [ ]
6. Which of the following have you undergone?
7. Male circumcision [ ]
8. Female Genital Mutilation [ ]
9. None of the above [ ]
10. Have you ever undergone blood transfusion at the hospital before

(a) Yes [ ]

(b) No [ ]

**Section 4 - Knowledge about HBV infection**

1. Have you ever heard of a disease called hepatitis B before?

(a) Yes [ ]

(b) No [ ]

1. If Yes, what causes hepatitis B disease

(a) Hepatitis B Virus [ ]

(b) Mosquitoes [ ]

(c) Housefly [ ]

1. Do you agree that, some of the ways by which one can get Hepatitis-B virus infection are (1) through infected mother to child (2) through sexual transmissions which is common among people with multiple sexual partners?

(a) Agree [ ]

(b) Strongly Agree [ ]

(c) Disagree [ ]

(d) Strongly Disagree [ ]

1. Do you also agree that one can be infected with Hepatitis B Virus through unsafe injections, blood transfusion, tattooing, scarification, and ear piercing?

(a) Agree [ ]

(b) Strongly Agree [ ]

(c) Disagree [ ]

(d) Strongly Disagree [ ]

1. Do you know that some of the signs and symptoms of Hepatitis B Viral Infection includes (1) yellowish eyes and skin (2) dark urine (3) abdominal pains (4) Vomiting?

(a) Yes [ ]

(b) No [ ]

1. Do you know that, Hepatitis B Virus infection may lead to the following complications (1) cirrhosis (2) liver cancer (3) and even death if not diagnosed early.

(a) Yes [ ]

(b) No [ ]

1. Do you agree that, Hepatitis B Virus infection can be prevented through the following measures (1) screening of blood for HBsAg before transfusion. (2) screening and vaccination of the at-risk adults as well as universal vaccination of neonate.

(a) Agree [ ]

(b) Strongly Agree [ ]

(c) Disagree [ ]

(d) Strongly Disagree [ ]

1. Do you know that Hepatitis B Virus infection can also be prevented through the following ways?

(1) Abstinence and use of condoms to reduce the risk of sexual transmission.

(2) Avoid sharing of sharps example blades and needles, sponge, toothbrush.

(a) Yes [ ]

(b) No [ ]

**Thank you.**
