# Supplementary material for: Seroprevalence and factors associated with Hepatitis B virus infection among students in two senior high schools in the Krachi Nchumuru district in Ghana-A cross-sectional study
Source: BMC Res Notes. 2023 Dec 2;16:358. doi: 10.1186/s13104-023-06624-4 (PMC10693693; doi:10.1186/s13104-023-06624-4)
Supplement: Supplementary file 2 — Additional file 2: Supplementary Data 1: Logistic Regression assumptions [file 13104_2023_6624_MOESM2_ESM.pdf]

Supplementary Data 1: Logistic Regression assumptions

```
. reg result spoon Needles shavingstick Blades shavingstick knife patnersnumber sexualrelationshi
> p condomuse tribalmarks tattoos earpeircing malecircumcision fgm bloodtransfusion
note: shavingstick omitted because of collinearity
note: sexualrelationship omitted because of collinearity
```

Source	SS	df	MS
Model	2.66048344	13	.204652572
Residual	20.5426416	178	.115408099
Total	23.203125	191	.12148233

Number of obs	=	192
F(13, 178)	=	1.77
Prob > F	=	0.0504
R-squared	=	0.1147
Adj R-squared	=	0.0500
Root MSE	=	.33972

result	Coef.	Std. Err.	t	P>|t|	[95% Conf.	Interval]
spoon	.0441309	.0621932	0.71	0.479	-.0786	.1668617
Needles	.0655167	.0544791	1.20	0.231	-.0419913	.1730247
shavingstick	-.0334826	.0607297	-0.55	0.582	-.1533253	.0863602
Blades	.0213462	.0545109	0.39	0.696	-.0862245	.1289169
shavingstick	0	(omitted)				
knife	.040334	.0672522	0.60	0.549	-.0923801	.1730482
patnersnumber	-.047508	.0399707	-1.19	0.236	-.1263855	.0313694
sexualrelationship	0	(omitted)				
condomuse	-.1135744	.0531728	-2.14	0.034	-.2185046	-.0086443
tribalmarks	.0423461	.0504092	0.84	0.402	-.0571303	.1418226
tattoos	-.0390147	.2474041	-0.16	0.875	-.5272372	.4492078
earpeircing	.1141108	.1195428	0.95	0.341	-.1217926	.3500143
malecircumcision	.3148254	.1138246	2.77	0.006	.0902061	.5394446
fgm	-.1120237	.2084833	-0.54	0.592	-.5234407	.2993932
bloodtransfusion	.0170679	.094289	0.18	0.857	-.1690002	.2031361
_cons	-.0713767	.1398258	-0.51	0.610	-.3473062	.2045528

```
. vif
```

Variable	VIF	1/VIF
earpeircing	5.94	0.168266
malecircum~n	5.34	0.187222
knife	1.43	0.699186
spoon	1.26	0.796640
Needles	1.23	0.811503
Blades	1.23	0.814808
patnersnum~r	1.21	0.827325
shavingstick	1.15	0.869224
fgm	1.11	0.899108
condomuse	1.09	0.917701
bloodtrans~n	1.07	0.938752
tribalmarks	1.06	0.946596
tattoos	1.05	0.952665
Mean VIF	1.86	

```
. swilk result malecircumcision earpeircing tattoos fgm bloodtransfusion tribalmarks condomuse pa
> tnersnumber
```

Shapiro-Wilk W test for normal data

Variable	Obs	W	V	z	Prob>z
result	300	0.96392	7.686	4.787	0.00000
malecircum~n	300	0.99978	0.048	-7.152	1.00000
earpeircing	300	0.99831	0.360	-2.397	0.99174
tattoos	300	0.99989	0.024	-8.779	1.00000
fgm	300	0.99989	0.024	-8.779	1.00000
bloodtrans~n	300	0.90442	20.363	7.074	0.00000
tribalmarks	300	0.99951	0.104	-5.308	1.00000
condomuse	192	0.99385	0.886	-0.278	0.60944
patnersnum~r	192	0.94194	8.360	4.876	0.00000

Supplementary Data 1: This supplementary information describes the output results for checking for multicollinearity and normality assumptions for logistic regression for our data. Variables that show collinearity were omitted in the regression and the data shows multivariate normality (Shapiro-Wilk W test).
